# Polymorphisms in chloroquine resistance-associated genes in *Plasmodium vivax* in Ethiopia

**DOI:** 10.1186/s12936-015-0625-3

**Published:** 2015-04-16

**Authors:** Lemu Golassa, Berhanu Erko, Frederick N Baliraine, Abraham Aseffa, Göte Swedberg

**Affiliations:** Aklilu Lemma Institute of Pathobiology, Addis Ababa University, Addis Ababa, Ethiopia; Department of Biology, LeTourneau University, Longview, TX USA; Armauer Hansen Research Institute, Addis Ababa, Ethiopia; Department of Medical Biochemistry and Microbiology, Uppsala University, Uppsala, Sweden

**Keywords:** Chloroquine resistance, Mutations, *Plasmodium vivax*, *Pvcrt*-o, *Pvmdr*-1

## Abstract

**Background:**

Evidence for decreasing chloroquine (CQ) efficacy against *Plasmodium vivax* has been reported from many endemic countries in the world. In Ethiopia, *P. vivax* accounts for 40% of all malaria cases and CQ is the first-line drug for *vivax* malaria. Mutations in multidrug resistance 1 (*pvmdr*-1) and K10 insertion in the *pvcrt*-o genes have been identified as possible molecular markers of CQ-resistance (CQR) in *P. vivax*. Despite reports of CQ treatment failures, no data are currently available on the prevalence of molecular markers of *P. vivax* resistance in Ethiopia. The objective of this study was to determine the prevalence of mutations in the *pvmdr*-1 and K10 insertion in the *pvcrt*-o genes.

**Methods:**

A total of 36 *P. vivax* clinical isolates were collected from West Arsi district in Ethiopia. Sequencing was used to analyse polymorphisms of the *pvcrt*-o and *pvmdr-*1 genes.

**Results:**

Sequencing results of the *pvmdr*-1 fragment showed the presence of two non-synonymous mutations at positions 976 and 1076. The Y → F change at codon 976 (TAC → TTC) was observed in 21 (75%) of 28 the isolates while the F → L change (at codon 1076), which was due to a single mutation (TTT → CTT), was observed in 100% of the isolates. Of 33 samples successfully amplified for the *pvcrt*-o, the majority of the isolates (93.9%) were wild type, without K10 insertion.

**Conclusions:**

High prevalence of mutations in candidate genes conferring CQR in *P. vivax* was identified. The fact that CQ is still the first-line treatment for vivax malaria, the significance of mutations in the *pvcrt*-o and *pvmdr*-1 genes and the clinical response of the patients’ to CQ treatment and whether thus an association exists between point mutations of the candidate genes and CQR requires further research in Ethiopia.

## Background

Of the five *Plasmodium* species infecting humans, *Plasmodium vivax* is the most widely distributed species and the cause of 25-40% of malaria cases worldwide [1], and substantial morbidity associated with vivax malaria has been reported [2-4]. Despite the public health importance, *P. vivax* malaria has received little attention and limited funds for research and control, since it usually produces less severe symptoms than falciparum malaria [2,5,6]. Current treatment for vivax malaria relies primarily upon two anti-malarial drugs, chloroquine (CQ) and primaquine (PQ), with the latter being the only effective drug against the hypnozoite stage. Indeed, the emergence of drug resistance in *P. vivax* particularly to the only class of compounds available for killing the dormant liver stage is alarming and of high priority for research [7-10]. It is worth noting that inadequate surveillance tools delayed the detection and containment of CQ-resistant *P. falciparum* resulting in increased morbidity and mortality. If a repetition is to be avoided, the threat of emerging CQ-resistant *P. vivax* needs to be acknowledged quickly and widely and substantial resources need to be allocated to validate and standardize tools necessary for characterization of drug-resistant *P. vivax* [10]. In Indonesia, East Timor and Papua New Guinea, CQ-resistant vivax malaria has already reached an alarming prevalence [11]. Furthermore, *P. vivax* CQR has occurred in at least three Latin American countries (Guyana, Peru and Brazil) [12]. The four clinical trials carried out in Asia (Thailand and Pakistan) and Africa (Ethiopia), for instance, showed that CQ alone (25 mg/kg over 3 days) is less effective against *P. vivax* asexual blood stages than CQ (25 mg/kg over 3 days) co-administered with PQ (15 mg of PQ base/day for 14 days) over 28 days of follow-up [9]. To unveil the current knowledge regarding the molecular mechanisms of *P. vivax* resistance to CQ and the prospects for developing and standardizing reliable molecular markers of drug resistance, Goncalves *et al.* [13] reviewed the available data by combining published *in vivo* and *in vitro* studies.

Unlike in *P. falciparum*, the molecular mechanism of *P. vivax* CQR remains elusive [7]. This is because, previous studies focusing on genes known to be main determinants of CQR in *P. falciparum* have failed to demonstrate a strong correlation between *pvcrt*-o and *pvmdr*-1 genotypes and the CQR phenotype in *P. vivax*. Melo *et al.* [14], on the other hand, showed the association of expression levels of *pvcrt*-o and *pvmdr*-1 with CQR and severe *P. vivax* malaria, because parasites from patients with CQR presented up to 6.1-fold and 2.4-fold increase in *pvcrt*-o and *pvmdr*-1 expression levels, respectively, compared to the susceptible group in the Brazilian Amazon.

Drug resistance in *P. vivax* is becoming more widespread, hindering management of clinical cases and posing a huge threat to the health of millions of people exposed to the risk of vivax malaria. Analysis of the single nucleotide polymorphisms (SNPs) in drug resistant genes has proved to be useful and important in monitoring drug resistance in malaria endemic countries [15]. Mutations in multidrug resistance 1 (*pvmdr*-1) and K10 insertion in the *pvcrt*-o genes have been identified as possible molecular markers of CQR in *P. vivax* [16,17]. Few data are available on the possible relationship between the *pvcrt*-o and *pvmdr-*1 genes and CQR [18]. Nevertheless, there are a number of contradicting reports regarding the association between *pvcrt*-o and *pvmdr*-1 polymorphisms and CQR. Some reports suggest the Y976F mutation in *pvmdr*-1 to be associated with an increase in CQ IC_50_ value of *P. vivax* isolates *in vitro* [19]. Non-synonymous amino acid mutations in codons Y976F and F1076L of the *pvmdr*-1 have been reported to have correlation with CQR although much work remains to link these mutations irrefutably with CQR [16,18,20]. The role of Y976F mutation in *pvmdr-*1 gene suggested reduced susceptibility to CQ [20]. Recent experiments have shown that the expression of *pvcrt*-o in transgenic lines of *P. falciparum* modulates CQ response [17]. A study by Fernandez-Becerra *et al.* [21] demonstrated up to 21-fold and up to three-fold increases in transcript levels of *pvcrt*-o and *pvmdr*-1, respectively, in severe vivax malaria cases compared to isolates from non-severe vivax malaria patients. Another study in India showed the predominance of the wild-type *pvmdr*-1 and *pvcrt*-o alleles [22] although one isolate had the Y976F mutation in the *pvmdr*-1 gene, which could suggest the beginning of a trend towards decreased CQ sensitivity. In Thailand and Indonesia, where CQR is common, the *pvmdr-*1 (Y976F and F1076L) polymorphisms were also identified in *P. vivax* samples [16]. In Latin America, where *P. vivax* CQR remains relatively uncommon, the Y976F and F1076L polymorphisms are relatively infrequent [23,24].

Presently, Ethiopia maintains a species-specific treatment policy: CQ without PQ is the first-line treatment for *P. vivax* and artemether-lumefantrine (AL) for *P. falciparum*. Unlike in many malaria endemic countries in Africa, both *P. falciparum* and *P. vivax* substantially contribute to malaria morbidity in Ethiopia in relative proportions of 60 and 40%, respectively [25,26]. In 1996, Ethiopia published its first report of CQR, with 2% (5/255) of study patients on CQ with persistent parasitaemia on day 7 [27] although 13% of treatment failures and subsequent reports CQR have been documented [28-30]. Indeed, data on the presence and prevalence of mutations in *pvmdr*-1 and *pvcrt*-o genes are limited in Ethiopia. The study was, therefore, initiated to determine the SNPs in the *pvmdr*-1 and *pvcrt*-o genes.

## Methods

### Study area, samples collection and diagnosis

The samples for this study were collected from West Arsi district from November to December 2012. Malaria transmission is seasonal and unstable in this area. Study participants were patients seeking malaria diagnosis at the Aje Health Centre, located at 0382146.3 E, 071734.2 N and 1,852 m above sea level. Malaria diagnosis was confirmed by microscopy of Giemsa-stained blood films and the species of *Plasmodium* were recorded. Finger-prick blood samples were collected from patients and used for thick and thin blood film preparation. Slides were considered negative after examination of 100 high-power fields. Patients showing positive results for *P. vivax* infection were treated with CQ. Blood samples spotted on filter paper were used for molecular analyses.

### Amplification and determination of *pvmdr*-1 and *pvcrt*-o polymorphisms

DNA was extracted from blood spots on filter paper using Chelex extraction methods as described elsewhere [31]. The *pvcrt*-o (K10 insertion) and *pvmdr*-1 (Y976F and F1076L) genes were amplified by nested PCR using gene-specific primers (Table 1). The outer and nested PCR conditions for *pvmdr*-1 was as follows: 94°C, 2 min; 33 cycles of 94°C, 15 sec; 56°C, 30 sec; 72°C, 1 min; 72°C, 7 min. The outer PCR condition was performed in 94°C, 2 min; 30 cycles of 94°C, 15 sec; 52°C, 30 sec; 72°C, 1 min; 72°C, 7 min, while the nested PCR was performed under the following conditions: 94°C, 2 min; 30 cycles of 94°C, 15 sec; 57°C, 30 sec; 72°C, 1 min; 72°C, 7 min. In both the *pvmdr*-1 and *pvcrt*-o loci, the nested forward as well as the reverse sequencing primer were used. PCR amplicons were analysed by nucleotide sequence determination at Uppsala Genome Center. Sequencing reactions were run with AB BigDye Terminator v3.1 and spin-column based clean-up. Sequencing samples were separated by capillary electrophoresis on the ABI3730XL DNA Analyzer (Applied Biosystems).

**Table 1:** **Primers used for amplifications of** ***pvcrt*** **-o and** ***pvmdr*** **-1 marker genes**

**Primers**	**Sequences 5′ → 3′**	**Size (bp)**
*Pvmdr-1* (OF)	CGCCATTATAGCCCTGAGCA	603
*Pvmdr-1* (OR)	TCTCACGTCGATGAGGGACT	
*Pvmdr-1* (NF)	GGATAGTCATGCCCCAGGATTG	
*Pvmdr-1* (NR)	CATCAACTTCCCGGCGTAGC	
*Pvcrt-o* (OF)	GCTACCCCTAACGCACAATG	253
*Pvcrt-o* (OR)	GATTTGGGAAAGCAGAACGT	
*Pvcrt-o* (NF)	GATGAACGTTACCGGGAGTTGG	
*Pvcrt-o* (NR)	ATCGGAAGCATCAGGCAGGA	
*Pvcrt-o* (Rseq)	GGGGACGTCCTCTTGTATTT	

OF (Outer forward), OR (outer reverse), NF (nested forward), NR (nested reverse).

### Ethical approval

Study protocol was reviewed and approved by Institutional Review Boards of Aklilu Lemma Institute of Pathobiology, Addis Ababa University and of the Armauer Hansen Research Institute as well as the National Research Ethics Review Committee.

### Data analysis

Since this study was a preliminary exploratory study, a power calculation of sample size was not done. Data were entered, validated and analysed in Microsoft Excel 2010. Allele proportions were calculated for codons of interest by dividing the number of samples with a particular allele to the number of samples with an identifiable allele at that position.

## Results

The *pvmdr-*1 gene was successfully amplified and sequenced in 78% (28/36) of the *P. vivax* isolates. Two *pvmdr*-1 mutant alleles were identified: Y976F alone and Y976F-F1076L. The prevalence of *pvmdr-*1 Y976F mutation was 75% (21/28) (Table 2). All (100%) isolates carried the *pvmdr-*1 F1076L mutation.

**Table 2:** ***pvmdr*** **-1 gene mutations and tandem repeat genotypes in** ***pvcrt*** **-o gene**

**Molecular markers**	**No. of isolates sequenced/no. of total isolate selected (%)**
***Pvmdr-1***	
Mutant Y976F	21/28 (75)
Mutant F1076L	28/28 (100)
***Pvcrt-o***	
Wild-type (without K10 insertion)	31/33 (93.9)

The *pvcrt*-o gene was successfully sequenced in 92% (33/36) of the isolates. Of the 33 samples successfully amplified for the *pvcrt*-o, the majority of the samples (93.9%) were wild type, without K10 insertion (Table 2). Synonymous mutations or insertions (in introns) were found in 6.1% (2/33) of the isolates.

## Discussion

CQ continues to be used for the treatment of *P. vivax* infection in Ethiopia despite reports of CQR from various studies in the country [28-30,32]. It is, therefore, important to investigate the prevalence of drug-resistance associated markers in *P. vivax* clinical isolates in this country. In *P. falciparum*, mutations in the *pfcrt* and *pfmdr*-1 genes have been linked to CQR but in *P. vivax* the picture is still unclear regarding the possible relationship between the *pvcrt*-o and *pvmdr*-1 genes and CQR. However, the Y976F substitution in the *pvmdr*-1 gene is thought to be involved in CQR in *P. vivax* [19] because the geometric mean 50% inhibitory concentration of CQ was shown to be significantly higher in *P. vivax* isolates carrying the Y976F mutation than in isolates with the wild-type allele. On the other hand, the ubiquitous presence of Y976F in all patients presenting to a clinic in Papua, where CQ resistance *P. vivax* is both at high and prevalent, precluded correlation with *ex vivo* drug susceptibility to CQ [10]. In the Thai isolates, the Y976F substitution was associated with a 1.7-fold higher IC50 to CQ [10]. Unlike the Y976F mutation, Suwanarusk *et al.* [20] found the *pvmdr*-1 F1076L mutation in all the isolates (wild type and mutants).

In the present study, 75% of the *P. vivax* isolates had the Y976F mutation in *pvmdr*-1. Sequencing results of the *pvmdr*-1 fragment showed the presence of two non-synonymous mutations at positions 976 and 1076. The Y → F change at codon 976 (TAC → TTC) was observed in 26 (75%) of 28 isolates. The second F → L change (at codon 1076), which was due to a single mutation (TTT → CTT), was observed in 100% of isolates. Whether isolates carrying the *pvmdr*-1 Y976F mutation responded to CQ treatments differently from those isolates with the wild-type sequence necessitates further *in vivo* therapeutic efficacy study in Ethiopia, but reports from Indonesia and Thailand suggest this to be the case [19]. The difference in the prevalence of *pvmdr-*1 Y976F in areas where CQR *P. vivax* prevails *versus* CQ remain efficacious may indicate the correlation between CQR and sequence polymorphisms in *pvmdr*-1. In Papua Indonesia, where CQR *P. vivax* is present at high prevalence (>65%) and high level [19], the *pvmdr*-1 Y976F mutation was present in all patients presenting to a clinic. In contrast, the sequence polymorphism in *pvmdr*-1 conferring Y976F was identified in only 25% of Thai isolates from an area where CQ remains efficacious. Ninety-six percent of Indonesian isolates (where clinical resistance to CQ prevails) had Y976F mutation, compared to 25% of Thai isolates where CQ sensitivity was almost uniform [19]. The fact that that all parasites with the Y976F substitution in Ethiopia also carried the F1076L mutation, as originally described by Brega *et al*. [22] the F1076L mutation could be a background mutation that precedes the Y976F substitution and could potentially provide an early warning on emerging CQR.

In Ethiopia, CQR *P. vivax* has been reported from various studies. Given the high prevalence of the Y976F mutations in *pvmdr*-1 in Southeast Asia where CQR prevails [19], the high prevalence of *pvmdr*-1 Y976F mutations identified in this study may be associated with the CQ treatment failure reported in Ethiopia. But the exact role of this mutation needs to be determined by a combination of *in vitro* and clinical observation studies in this country. The fact that all isolates carried the *pvmdr*-1 F1076L mutation, substitution in this codon may be less involved in the modulation of *P. vivax* susceptibility to CQ than the *pvmdr*-1 Y976F mutation given that CQ is still effective and widely used in Ethiopia. Indeed, the presence of *pvmdr*-1 F1076L mutation in all susceptible and mutant isolates challenged the role of *pvmdr*-1 polymorphisms in modulating CQ responses in *P. vivax* [33]. On the other hand, *pvmdr*-1 polymorphisms have been recently suggested to be associated with CQR in Southeast Asia [19] unlike polymorphisms in *pvcrt*-o that have not been associated with CQR in *P. vivax*. The limitation of this study was that it did not determine drug resistance phenotype (either *in vivo* or *in vitro*) for the isolates undergoing molecular characterization at the *pvmdr-*1 and *pvcrt*-o genes. Indeed, withdrawal of a given drug is recommended when 10% of infections are not responding to treatment, although in practice, governments of poor countries leave it longer [34]. The fact that CQ treatment failure reported earlier in Ethiopia did not exceed the level to withdrawal CQ, periodic assessment of the current status of CQR *P. vivax* has great public health significance**.**

## Conclusion

Despite the fact that *P. vivax* accounts for about 40% of malaria cases, little attention has been given to the urgent public health need to detect and to closely monitor the progression of CQ-resistant vivax malaria in the country. This study has observed a high prevalence of the *pvmdr*-1 976 F allele, which is believed to be associated with CQR in *P. vivax*. In view of reports from elsewhere, the high prevalence of *pvmdr*-1 Y976F mutation identified in this study may be associated with the reported CQ treatment failure Ethiopia. However, determination of the exact role of this particular mutation in *P. vivax* CQ responses, as well as the roles of other identified *pvmdr*-1 and *pvcrt*-o gene mutations needs further research, involving a combination of *in vitro* and clinical observation studies in this country.
