# Correction to: Polymorphisms in chloroquine resistance-associated genes in *Plasmodium vivax* in Ethiopia

**DOI:** 10.1186/s12936-018-2338-x

**Published:** 2018-05-02

**Authors:** Lemu Golassa, Berhanu Erko, Frederick N. Baliraine, Abraham Aseffa, Göte Swedberg

**Affiliations:** 1 Aklilu Lemma Institute of Pathobiology, Addis Ababa University, Addis Ababa, Ethiopia; 2 Department of Biology, LeTourneau University, Longview, TX USA; 3 Armauer Hansen Research Institute, Addis Ababa, Ethiopia; 4 Department of Medical Biochemistry and Microbiology, Uppsala University, Uppsala, Sweden

## Correction to: Malaria J (2015) 14:164 10.1186/s12936-015-0625-3

After publication of the original article [1], it came to the authors’ attention that the primers mentioned in Table 1 for the amplification of the *pvcrt*-*o* gene of *Plasmodium vivax* are not the ones actually used for the experiments. The correct primers and PCR product size are as below:

**Table 1:** Primers used for amplifications of *pvcrt*-o and *pvmdr*-1 marker genes

PCR	Sequences 5´ → 3´
Outer forward (OF)	CCA GTG AGA AGC CCC TGT TC
Outer reverse (OR)	CGA AGT ATG CGC GTG AAG
Nested forward (NF)	AAG AGC CGT CAA GCC ATC
Nested reverse (NR)	AGT TTC CCT CTA CAC CCG
*pvcrt*-*o* (Rseq)	GGG GAC GTC CTC TTG TAT TT

*OF* Outer forward, *OR* outer reverse, *NF* nested forward, *NR* nested reverse. Size (nested) 1190 bp

## References

[1] Golassa L, Erok B, Baliraine FN, Aseffa A, Swedberg G (2015). Polymorphisms in chloroquine resistance-associated genes in *Plasmodium vivax* in Ethiopia. Malaria J.

